# Correlating anterior insula gray matter volume changes in young people with clinical and neurocognitive outcomes: an MRI study

**DOI:** 10.1186/1471-244X-12-45

**Published:** 2012-05-20

**Authors:** Sean N Hatton, Jim Lagopoulos, Daniel F Hermens, Sharon L Naismith, Maxwell R Bennett, Ian B Hickie

**Affiliations:** 1 Clinical Research Unit, Brain & Mind Research Institute, University of Sydney, Sydney, NSW, Australia; 2 Postal Address: Brain and Mind Research Institute, University of Sydney, 100 Mallet Street, Sydney, NSW, 2050, Australia

**Keywords:** Insula, Depression, Anxiety, Bipolar, Psychosis, MRI, Symptoms, Executive function

## Abstract

**Background:**

The anterior insula cortex is considered to be both the structural and functional link between experience, affect, and behaviour. Magnetic resonance imaging (MRI) studies have shown changes in anterior insula gray matter volume (GMV) in psychosis, bipolar, depression and anxiety disorders in older patients, but few studies have investigated insula GMV changes in young people. This study examined the relationship between anterior insula GMV, clinical symptom severity and neuropsychological performance in a heterogeneous cohort of young people presenting for mental health care.

**Methods:**

Participants with a primary diagnosis of depression (*n* = 43), bipolar disorder (*n* = 38), psychosis (*n* = 32), anxiety disorder (*n* = 12) or healthy controls (*n* = 39) underwent structural MRI scanning, and volumetric segmentation of the bilateral anterior insula cortex was performed using the FreeSurfer application. Statistical analysis examined the linear and quadratic correlations between anterior insula GMV and participants’ performance in a battery of clinical and neuropsychological assessments.

**Results:**

Compared to healthy participants, patients had significantly reduced GMV in the left anterior insula (*t* = 2.05, *p* = .042) which correlated with reduced performance on a neuropsychological task of attentional set-shifting (*ρ* = .32, *p* = .016). Changes in right anterior insula GMV was correlated with increased symptom severity (*r* = .29, *p* = .006) and more positive symptoms (*r* = .32, *p* = .002).

**Conclusions:**

By using the novel approach of examining a heterogeneous cohort of young depression, anxiety, bipolar and psychosis patients together, this study has demonstrated that insula GMV changes are associated with neurocognitive deficits and clinical symptoms in such young patients.

## Background

Located between the frontal, temporal and parietal lobes, the insular cortex processes a vast array of information including visceral sensory, visceral motor, vestibular, pain, temperature, language, visual, auditory, and tactile information [1,2]. A meta-analysis of 162 functional neuroimaging studies [3] highlights that the anterior insula is consistently activated during the expression of emotion including anger [4], sadness [4], fear [5], disgust [6], happiness or joy [7-9], trust [10], and surprise [11], as well as social emotions [reviewed in [12]]. Furthermore, the anterior insula has been implicated in visual-tactile [13] and auditory-visual [14] integration, as well as olfactory and gustatory function [15-17], suggesting that the insular cortex integrates these special visceral senses with behavioural and emotional events [18,19]. These processes arise through elaborate and extensive structural connections between the insula and various cortical, basal nuclei, limbic and thalamic areas [reviewed in [20]]. Thus, the insular cortex is considered to be the structural and functional link between experience, affect, and behaviour [21].

Functional network analysis suggests that the insular cortex processes information through high-level cognitive control and attentional processes, mediating “salience switching” between other large functional networks involved in externally-oriented attention and internally-oriented cognition [reviewed in [22]]. Functional magnetic resonance imaging (fMRI) studies have reported that the bilateral anterior insula, together with the anterior cingulate cortex, show increased activation during working memory, target switching and cognitive control tasks [23,24]. Furthermore, an fMRI study on attentional transitioning found that the bilateral anterior insula and anterior cingulate cortex operate as a cognitive-control network, and that the right anterior insula plays an essential role of switching between the central executive network and the default-network [25]. Cumulatively, the insular cortex, and in particular the anterior insula, appears to mediate the processing of experience, affect and behaviour [21] via high-level executive functioning and salience switching [22].

Changes in insula GMV have been implicated in the aetiology of many common psychiatric disorders in older patients. Meta-analysis of magnetic resonance imaging (MRI) studies has demonstrated significant insula GMV loss in bipolar disorders [26-29] and psychosis [26,30-34], and to a lesser extent in anxiety disorders [35-37]. The findings relating to unipolar depression appear to be less consistent, with some demonstrating no association [38-41], and others showing insula GMV loss in patients with depression [42,43]. However, as the interpretation of insula changes in older patients is confounded by long illness durations, associated lifestyle factors and up to 20 years of medication use, it is important to examine if changes in anterior insula GMV is evident in young patients at the early stages of disease progression. A voxel-based morphometry (VBM) meta-analysis [44] summarised that, in comparison to healthy controls, young subjects at enhanced clinical risk for psychosis exhibited reduced GMV in the left insular cortex. A meta-analysis of MRI abnormalities in young patients with bipolar disorder [45] reported that most studies found gray matter loss in the subgenual, prefrontal and temporal regions, though findings between studies were inconsistent. Lagopoulos et al. [46] conducted a cross-sectional VBM analysis of 47 young people with a heterogeneous mixture of depressive and psychosis symptoms, finding that compared to healthy controls, patients at early stages of illness had GMV reductions in the left insula, and this GMV loss was exacerbated in patients at later stages of disease progression. Examining changes within the insula of young people with emerging disorders would thus inform models of pathophysiology that link underlying neurobiological changes with key symptoms, helping to inform early detection and targeted intervention strategies [47-50].

In this study, we therefore aimed to determine whether: a) young people with an emerging anxious, affective or psychotic illness demonstrate evidence of anterior insular change, relative to their age-matched controls; and b) changes in the anterior insula relate to clinical symptoms and neuropsychological performance, specifically in the domain of executive functioning.

## Methods

### Participants

One hundred and thirty three outpatients aged 16 to 30 years were recruited from specialist youth mental health clinics in Sydney Australia [51,52]. Thirty-nine healthy controls were recruited from the community in the same region, and were screened for history of psychiatric disorders.

Exclusion criteria for both patients and controls were medical instability (as determined by a psychiatrist), history of neurological disease (e.g. tumour, head trauma, epilepsy), medical illness known to impact cognitive and brain function (e.g. cancer), intellectual and/or developmental disability, insufficient English for neuropsychological assessment and current substance dependence. All participants were asked to abstain from drug or alcohol use for 48 hours prior to testing and informed about a drug screen protocol. To verify recent abstinence, participants underwent an alcohol breath test and a saliva drug screen to determine presence of cannabinoids, meth/amphetamines, opiates, benzodiazepines and cocaine. The study was approved by the University of Sydney ethics committee. Participants gave written informed consent prior to participation in the study.

Patients were determined to have a primary diagnosis of depression (*n* = 43), bipolar disorder (*n* = 38), psychosis (*n* = 32) or anxiety disorder (*n* = 12) by a psychiatrist, according to DSM-IV-TR criteria [53]. At the time of assessment, 15% of patients were not taking any psychotropic medications; 51% were taking a second-generation antidepressants, 42% an atypical antipsychotic medication, 7% were taking a mood stabiliser and 2% were taking stimulants. Of those medicated, 48% were taking more than one of these psychotropic medications; for the majority of these patients (40% of those medicated) this polytherapy included an anti-psychotic.

### Clinical assessment

All participants underwent clinical and neuropsychological assessment as previously described [54,55]. A psychiatrist conducted the clinical assessment (in a semi-structured interview format) to inform the diagnostic classification and to determine the nature and history of any mental health problems. Participants’ educational level was assessed as the cumulative completed number of years in school, university and/or advanced diploma course. The assessment included the Social and Occupational Functioning Assessment scale [SOFAS; [56]] to assess general social and occupational functioning, the Hamilton Depression Rating Scale [HDRS, 17-item; [57]] to quantify current (over the last 7 days) mood symptoms, and the Brief Psychiatric Rating Scale [BPRS; [58]] to quantify current general psychiatric symptom severity. The 24-point BPRS Total score is further subtyped by subscores assessing depression (somatic concern, anxiety, depression, suicidality, guilt, self-neglect), positive symptoms (hostility, grandiosity, suspiciousness, hallucinations, unusual thought content, bizarre behaviour, conceptual disorganization), negative symptoms (self-neglect, blunted affect, emotional withdrawal, motor retardation, uncooperativeness), mania (elated mood, conceptual disorganisation, tension, uncooperativeness, excitement, distractibility, motor hyperactivity) and disorientation (disorientation, mannerisms & posturing). As the BPRS disorientation subscore comprises of only two items, the measure was not considered statistically robust and was omitted from this study.

### Self-report

All participants completed a self-report assessment that included: the Kessler-10 [K-10; [59]] which is a brief instrument designed to detect psychological distress [60]; the Depression Anxiety and Stress Scales [DASS; [61]], which measures the three related negative emotional states of depression, anxiety, and tension/stress; and, the Social Interaction Anxiety Scale [SIAS; [62]] which measures social interaction anxiety.

### Neuropsychological assessment

As part of a broader battery [described previously, [54,55,63]], a trained research psychologist administered standardised tests to examine the following functions:

a) Premorbid IQ: This was estimated from the Wechsler Test of Adult Reading [64] where participants are scored on their ability to pronounce 50 English language words of increasing complexity.

b) Mental flexibility: The Trail-Making Test part B assessed “mental flexibility” [TMT-B; [65]], whereby participants were required to draw a line connecting consecutive targets alternating between numbers and letters. Raw timed scores were converted to age-adjusted *z*-scores according to normative date [66].

c) Verbal fluency: This was assessed via a subtest of the Controlled Oral Word Association Test [COWAT; [65]] which assessed the generation of words starting with the letters F, A and S, within a one-minute interval. Age- and educational-adjusted *z*-scores were derived from normative data [66].

d) Set-shifting: This was measured using the Intra-Dimensional/ Extra-Dimensional task (IED) from the Cambridge Automated Neuropsychological Testing Battery [CANTAB; [67]]. The total error score (adjusted) was used, measuring the subject’s efficiency to attend to specific attributes of compound stimuli (e.g. a line and a shape), then shift attention from one attribute (e.g. select the shape) to another (e.g. now select the line).

e) Sustained attention: This was measured via the Rapid Visual Information Processing task (RVP-A), of the CANTAB [67]. The A prime measure was used, reflecting the participant’s accuracy at detecting a target sequence of numbers (e.g. 4-5-3) within 2 minutes of pseudo-random numbers (e.g. 3-6-9-**4-5-3**-7- etc.).

f) Spatial working memory: This was assessed by the longest sequence length of patterns recalled in the Spatial Span (SSP) task of the CANTAB [67].

### Magnetic resonance imaging acquisition and analysis

Participants underwent structural MRI scanning using a 3-Tesla GE scanner at Southern Radiology MRI Diagnostic Services within the Brain and Mind Research Institute, Camperdown, NSW Australia. The images where acquired using a customized MP-RAGE 3D T1-weighted sequence to resolve anatomy at high resolution (0.9mm isotropic resolution); TR = 7264msec; TE = 2784msec; pulse angle = 15; coronal orientation; FOV 230 mm^3^; matrix of 256 x 256 x 196.

Volumetric segmentation was performed with the FreeSurfer application version 5.1 (http://surfer.nmr.mgh.harvard.edu/) and technical details of these procedures have been previously described [68-78]. Briefly, this process involves the motion correction and averaging of two volumetric T1-weighted images [79], removal of non-brain tissue [78], transformation of the scans to the standard Talairach space, segmentation of the subcortical white matter and deep gray matter volumetric structures [71,72], intensity normalization [80], tessellation of the gray matter/white matter boundary, topology correction [70,81], surface deformation along intensity gradients to optimally place the gray/white and gray/cerebrospinal fluid borders [68,69,82], and finally cortical representations were parcellated into anatomical structures. Results were visually inspected and any inaccuracies were manually edited. FreeSurfer’s procedure for measuring cortical thickness have been validated against histological analysis [83] and manual measurements [84,85], and morphometric procedures have been demonstrated to show good test-retest reliability across scanner and field strengths [76].

Several structures clearly delineate the anterior insular cortex, as described in the Destrieux atlas and utilised in the FreeSurfer segmentation process [86]. The circular sulcus of the insula demarcates the insula along three boarders; the anterior segment separates the insula from the orbital gyri, the superior segment separates the insula from the opercular segment of the inferior frontal gyri, and the inferior segment separates the insula from the superior aspect of the superior temporal gyrus. Finally the central sulcus of the insula isolates the posterior insula (long insular gyrus) from the anterior insula (short insular gyri).

The intracranial volume (ICV) was measured to correct for differences in head size [87]. Region of interest (ROI) volumes were then corrected for ICV variation so as to provide a common space for cross-sectional morphometric comparisons.

### Statistical analysis and data transformations

Statistical analyses were performed using the Statistical Package for the Social Sciences (SPSS 19.0 for Mac). ROI outliers beyond a standard deviation of ±3.0 were removed from analysis. With the exception of the Wechsler Test of Adult Reading (“predicted IQ”), all neuropsychological scores were then converted to a standardised score (i.e. *z*-score) and outliers beyond a standard deviation of ±2.5 were curtailed to values of +2.5 or −2.5 (depending on the direction), enabling a consistent range across variables as previously described [54,88].

Between groups (healthy, patient) analyses were conducted using independent-sample *t*-tests, or chi-square tests for categorical data. Pearson partial correlations (*r*) explored the linear correlation between the left or right anterior insula GMV (corrected for ICV) with patient demographics, class of medication dosage (mg/day), clinical scores and neurocognitive performance controlling for age (*n* = 133). Due to the small number of patients using stimulant medication (*n* = 3), correlations between stimulants and GMV was omitted. Spearman’s correlation (*ρ*) was used when data violated the assumptions of normality and/or contained influential data points. Follow-up analysis repeated this process controlling for years of education.

Curve estimation regression explored the quadratic correlation between the left or right anterior insula GMV (ICV corrected) with the same patient variables described above (*n* = 133). Curve estimation fits a quadratic curve to the existing data points to examine if there is a “U-curve” pattern to the trend, inferring that the same outcome occurs when there is any departure from normality. This study employed quadratic correlation to see if any changes in anterior insula GMV (i.e. either increases or decreases from the norm) resulted in worse clinical symptom severity and/or neurocognitive performance. Correlations were considered significant where either *r* ≥ .30 or *ρ* ≥ .30 (medium effect, explains 9% of the total variance) and *p* < .05 (2-tailed).

## Results

### Demographics

As shown in Table 1, comparison between patients and healthy participants revealed no significant difference in the distribution of gender nor predicted IQ, however patients were significantly younger [*t*(95.2) = 2.95, *p* = .004] and less educated [*t*(161) = 33.08, *p* < .001)] compared to control subjects.

**Table 1:** Demographics and clinical symptom scores (± standard deviation)

**Demographic variable/clinical symptom score**	**Healthy (*n*** **= 39)**	**Patients (*n*** **= 133)**	**Significance test**
Female, percentage (f/m)	66.7% (26/13)	62.4% (83/50)	χ^2^(1, *n* = 172) = 0.24
Age, years^#^	23.8 ± 2.4	22.3 ± 3.7	*t*(95.2) = 2.95^**^
Predicted IQ	105.3 ± 8.2	104.1 ± 8.2	*t*(145) = 0.73
Education, years	15.1 ± 1.9	12.6 ± 2.4	*t*(161) = 33.08^**^
SOFAS		61.6 ± 12.5	
K-10 Total		27.6 ± 8.0	
HDRS Total		14.1 ± 7.4	
BPRS Total		42.3 ± 10.2	

Significant differences in gender were evaluated using a Pearson Chi-square test. Independent samples *t*-tests examined differences in age, predicted IQ and years of education. ^**^p < .01 (2-tailed) ^#^As Levene’s test for equality of variance was significant (*p* < .05) for age, results that did not assume equal variance were used.

### Anterior insula GMV differences between patients and healthy participants

Table 2 shows the left and right anterior insular volumes for patients and healthy participants. The left anterior insula was significantly reduced in patients compared to healthy participants [*t*(170) = 2.05, *p* = .042], but there was no significant right anterior insula GMV difference between groups [*t*(79.1) = 1.85, *p* = .069].

**Table 2:** Mean anterior insula gray matter volumes by cohort

**Region of interest**	**Healthy mean volume (mm**^**3**^ **± SD)**	**Patient mean volume (mm**^**3**^ **± SD)**	**Mean difference (mm**^**3**^ **± SE)**	**Significance test**
Left anterior insula	2209 ± 214.2	2124 ± 232.2	85 ± 41.6	*t*(170) = 2.05^*^
Right anterior insula	2019 ± 182.4	1953 ± 236.1	66 ± 35.7	*t*(79.1) = 1.85

Independent samples *t*-tests examined differences between patients (*n* = 133) and healthy participants (*n* = 39) in anterior insula volumes (corrected for ICV). As Levene’s test for equality of variance was significant (*p* < .05) for the right anterior insula volumes, results that did not assume equal variance were used. ^*^p < .05 (2-tailed). SD, standard deviation; SE, standard error.

### Relationship between demographics, clinical presentation and anterior insula size

Analysing patients only, there was no linear correlation found (i.e. *r* < .30 or *ρ* < .30) between anterior insula size and either demographics (predicted IQ, age of onset of illness, years of education), medication dosage (antidepressant, antipsychotic, mood stabiliser) or clinical symptom scale scores (SOFAS, K-10, HDRS, DASS, SIAS and BPRS measures) when controlling for age (Table 3). A follow-up analysis controlling for years of education did not find any significant linear correlations (see Additional file 1).

**Table 3:** Linear correlations of demographics and clinical variables to patient anterior insula GMV

**Demographic/clinical variable**	**Left anterior insula**	**Right anterior insula**
Predicted IQ	*r*(117) = .03	*r*(117) = −.01
Age of onset of illness	*r*(124) = −.02	*r*(124) = −.04
Years of Education	*r*(125) = −.10	*r*(125) = −.05
Antidepressant dose (mg/day)	*r*(110) = .09	*r*(110) = .02
Antipsychotic dose (mg/day)	*r*(110) = −.04	*r*(110) = −.10
Mood stabiliser dose (mg/day)	*r*(110) = .03	*r*(110) = .04
Social and occupational functioning (SOFAS)	*r*(119) = −.12	*r*(119) = −.11
Psychological distress (K-10)	*r*(125) = .08	*r*(125) = .06
Depression (HDRS)	*r*(121) = .10	*r*(121) = −.04
Depression (DASS Depression subscore)	*r*(116) = .11	*r*(116) = .00
Anxiety (DASS Anxiety subscore)	*r*(116) = −.01	*r*(116) = −.03
Stress (DASS Stress subscore)	*r*(116) = .08	*r*(116) = .03
Social interaction anxiety (SIAS)	*r*(122) = .18^*^	*r*(122) = .23^**^
Symptom severity (BPRS Total)	*r*(116) = .12	*r*(116) = .03
Positive symptoms (BPRS Positive Symptoms subscore)	*r*(116) = .10	*r*(116) = .10
Negative symptoms (BPRS Negative Symptoms subscore)	*r*(116) = .12	*r*(116) = .02
Depression (BPRS Depression subscore)	*r*(116) = .01	*r*(116) = −.09
Mania (BPRS Mania subscore)	*r*(116) = .08	*r*(116) = .06

Pearson partial correlations explored the linear correlations between the left or right anterior insula GMV (corrected for ICV) with patient demographic and clinical variables (*n* = 133) controlling for age. ^*^p < .05; ^**^p < .01 (2-tailed).

Quadratic correlation analysis of demographics, medication dosage, clinical symptoms and anterior insula size found that changes in anterior insula GMV were associated with increased psychiatric symptom severity, as measured by the BPRS score (Table 4). There was a significant correlation between BPRS Total score and the right anterior insula size [*r*(117) = .29, *p* = .006]. Similarly, there was a significant correlation between BPRS positive symptoms subscore and the right anterior insula size [*r*(117) = .32, *p* = .002].

**Table 4:** Quadratic correlations of demographics and clinical variables to patient anterior insula GMV

**Demographic/clinical variable**	**Left anterior insula**	**Right anterior insula**
Predicted IQ	*r*(117) = .03	*r*(117) = .04
Age of onset of illness	*r*(124) = .07	*r*(124) = .09
Years of Education	*r*(125) = .16	*r*(125) = .13
Antidepressant dose (mg/day)	*r*(64) = .13	*r*(64) = .06
Antipsychotic dose (mg/day)	*r*(60) = .20	*r*(60) = .26
Mood stabiliser dose (mg/day)	*r*(25) = .25	*r*(25) = .17
Social and occupational functioning (SOFAS)	*r*(119) = .12	*r*(119) = .13
Psychological distress (K-10)	*r*(125) = .12	*r*(125) = .10
Depression (HDRS)	*r*(121) = .17	*r*(121) = .19
Depression (DASS Depression subscore)	*r*(116) = .13	*r*(116) = .02
Anxiety (DASS Anxiety subscore)	*r*(116) = .14	*r*(116) = .13
Stress (DASS Stress subscore)	*r*(116) = .19	*r*(116) = .09
Social interaction anxiety (SIAS)	*r*(122) = .20	*r*(122) = .28^**^
Symptom severity (BPRS Total)	*r*(117) = .17	*r*(117) = .29^**^
Positive symptoms (BPRS Positive Symptoms subscore)	*r*(117) = .17	*r*(117) = .32^**^
Negative symptoms (BPRS Negative Symptoms subscore)	*r*(117) = .10	*r*(117) = .06
Depression (BPRS Depression subscore)	*r*(117) = .07	*r*(117) = .10
Mania (BPRS Mania subscore)	*r*(116) = .16	*r*(116) = .22

Curve estimation regression explored the quadratic correlations between the left or right anterior insula GMV (corrected for ICV) with patient demographic and clinical variables (*n* = 133). ^**^p < .01 (2-tailed).

Quadratic correlation analysis also highlights a significant correlation whereby changes in anterior insula GMV were associated with worse social anxiety as measured by the SIAS score (Table 4). There was a significant association between SIAS score and the right anterior insula size [*r*(122) = .28, *p* = .006]. Other variables analysed did not exhibit significant correlations.

### Relationship between executive functioning and anterior insula size

Linear correlation analysis of executive function found that there was a positive association between anterior insula GMV and ability to perform an attentional set-shifting task (Table 5). Specifically, there was a significant correlation between IED Total errors and left anterior insula size [*ρ*(109) = .32, *p* = .016]. To confirm that performance in the IED test was not influenced by either levels of education or IQ, follow-up linear correlation analysis found that IED performance was not significantly associated with either years of education [*ρ*(108) = .027, *p* = .783] or predicted IQ [*ρ*(107) = .175, *p* = .071]. There were no significant linear correlations with the remaining measures of executive function, namely the SSP, TMT-B, RVP-A and COWAT measures. A follow-up analysis controlling for years of education found similar results; the only significant correlation was found between IED Total errors and left anterior insula size [*ρ*(109) = .32, *p* = .001; see Additional file 1].

**Table 5:** Linear and quadratic correlations of neurocognitive executive functioning to patient anterior insula GMV

	**Linear correlation**		**Quadratic correlation**	
**Neurocognitive performance**	**Left anterior insula**	**Right anterior insula**	**Left anterior insula**	**Right anterior insula**
Set Shifting (IED Total error)	*ρ*(109) = .32^**^	*ρ*(109) = .13	*r*(106) = .31^**^	*r*(106) = .22
Working Memory (SSP)	*ρ*(107) = .08	*ρ*(107) = −.00	*r*(104) = .20	*r*(104) = .16
Mental Flexibility (TMT-B)	*ρ*(114) = −.04	*ρ*(114) = .04	*r*(111) = .02	*r*(111) = .10
Sustained Attention (RVP-A)	*r*(106) = −.08	*r*(106) = −.05	*r*(103) = .13	*r*(103) = .05
Verbal Fluency (COWAT)	*r*(112) = .10	*r*(112) = .09	*r*(109) = .10	*r*(109) = .14

Spearman’s *ρ* (non-normal score distribution) or Pearson’s *r* (normal score distribution) correlation analysis explored the linear relationship between the left or right anterior insula GMV (corrected for ICV) against *z*-scores of neuropsychological assessments of executive function (*n* = 133), controlling for age. Curve estimation regression explored the quadratic correlation between the same regions of interest and neuropsychological assessments of executive function. ^**^p < .01 (2-tailed).

Quadratic correlation analysis of executive function found a similar positive correlation between anterior insula GMV and the IED Total errors score (Table 5). There were significant correlations between the number of IED Total errors and left anterior insula size [*r*(106) = .31, *p* = .005]. There were no significant quadratic correlations with the remaining measures of executive function.

## Discussion

The present study sought to investigate anterior insula GMV changes in a heterogeneous sample of young psychiatric patients, and if such GMV changes related to severity of clinical symptoms or impairments in neurocognitive executive functioning. This study revealed that compared to age-matched healthy participants, young psychiatric patients have reduced left anterior insula GMV that positively correlated with neurocognitive performance in an attentional set-shifting task. GMV loss or gain in the right anterior insula was associated with increased general psychiatric symptom severity (BPRS Total score) and increased positive symptoms (BPRS Positive Symptoms subscore).

The major finding from this study is that young people with emerging mental disorders have reduced left anterior insula GMV that correlates with worse executive functioning (Tables 2,5), in particular, set-shifting. The asymmetry of a larger left insula relative to the right insula is most apparent in humans, with less hemispheric difference seen in similar-sized lower primates [89], and this feature is hypothesised to be due to the left insula’s role in human language and language-associated functions [20]. This present study’s findings that left anterior insula GMV is positively correlated with attentional set-shifting (IED) but not verbal fluency (COWAT), working memory (SSP) or sustained attention (RPV-A) suggests that the left anterior insula may be more closely involved in attentional processing rather than vocabulary memorisation. Interestingly, attention is conspicuously affected in all psychiatric disorders [90], and hence anterior insula degradation may be a core biological marker in this neurocognitive deficit. This result reinforces the “salience network” hypothesis [22,25] that the anterior insula is involved in modulating reactivity to salient internal and extrapersonal stimuli, and this attentional executive function is impaired in young psychiatric patients with a broad variety of disorders.

The modulation of altered afferent interoceptive input, self-referential and belief-based states, and poorly predictive signals has been proposed as a neuroanatomical model for depression and anxiety [91]. One model of anxiety suggests that neurons in the anterior insula estimate an “interoceptive prediction error” which signals a mismatch between anticipated and actual bodily responses to a potentially aversive stimulus [92]. Studies demonstrating increased insula activation during emotion processing in depressive and anxiety-prone individuals [91,93] strengthens the argument that degradation of the anterior insula might result in maladaptive motivating signal for individuals to withdraw (depression) or avoid (anxiety) situations. This study found there was no significant relationship between clinical measures of general anxiety (DASS) or depression (HDRS, DASS, BPRS depression subscore) and changes in anterior insula GMV (Tables 3 and 4), highlighting that anterior insula degradation in depression and anxiety may be a functional dysregulation rather than a structural alteration.

Both increases and decreases in right anterior insula GMV were associated with more positive symptoms (Table 4), namely hostility, grandiosity, suspiciousness, hallucinations, unusual thought content, bizarre behaviour, and conceptual disorganization. It has been suggested that since the insular cortex provides connections between the limbic memory regions and sensory systems, altered insula function may result in unbalanced sensory-memory integration, such as sensory hallucinations [94]. Indeed, an fMRI study [95] of psychotic patients found auditory hallucinations co-occurred with significant hyperactivity within the right anterior insula and to a lesser extent the left insula. One structural MRI paper investigating GMV changes in schizophrenia [96] found that schizophrenic patients with hallucinations had reduced bilateral insula GMVs when compared to healthy controls, and reduced left insula GMVs when compared to schizophrenic patients without hallucinations. This present study demonstrates that in younger patients, positive symptoms are related with any imbalance of the right anterior insula GMV, consistent with the hypothesis of a hemispheric imbalance of salience switching [22,25] which may underlie dysfunctional sensory awareness and interpretation [97].

Changes in right anterior insula GMV were associated with increased clinical symptom severity as measured by the BPRS Total score (Table 4). Other clinical scales employed in this study specifically targeting depression (DASS, HDRS), anxiety (DASS), psychological stress (K-10, DASS) and social functioning (SOFAS) were not correlated with anterior insula GMV changes. Considering that the BPRS Total score encompasses a depression subscore, the significant result of the BPRS Total score is probably due to the positive symptom subscore result discussed above. However, since the BPRS Total score is the summation of 24 questions it might be considered to be the largest, most robust measure of symptom severity in this study. Accordingly, the BPRS Total score may be a robust measure of symptom severity in young people arising from changes in right anterior insula GMV.

The relationship between anterior insula GMV and clinical measures of social anxiety remains inconclusive (Tables 3,4). The SIAS self-report assesses fears of social interaction situations and has been demonstrated to discriminate people diagnosed with social anxiety disorder from healthy controls [98] and other anxiety disorders [62]. In this study the SIAS score had a weak but significant positive linear correlation with the right anterior insula GMV (Table 3), suggesting that reductions in anterior insula GMV equate to reductions in social anxiety and vice-versa. Future research could further investigate associations between anterior insula GMV changes and social anxiety severity using a larger cohort of patients in combination with other anxiety scales such as the clinician-administered Liebowitz Social Anxiety Scale [LSAS; [99]], the consumer-supported Social Phobia and Anxiety Inventory [SPAI; [100,101]], or the refined self-report SIAS [102] with the companion Social Phobia Scale [SPS; [62]].

The influence of medication on changes in GMV remains contentious. Antidepressants, antipsychotics and mood stabilisers can be neuroprotective in patients and animal models of psychiatric disorders [reviewed in [103]]. A recent meta-analysis [104] suggest that antipsychotic medication may contribute to some structural changes noted in psychosis patients, though the authors concede that this may be confounded by disease progression [105]. A ROI MRI study of psychosis patients [106] found that insula gray matter volume was not influenced by antipsychotic medication, and a follow-up study with bipolar patients [107] found that insula volume was not affected by lithium or valproate medication. In the young patients examined in this study, there was no evidence suggesting a relationship between antidepressants, antipsychotics or mood stabilisers with anterior insula GMV changes. Future research could examine how anterior insula GMV may be affected by specific medications, as well as the impact of medication switching over time.

Future studies can enhance the findings of this present cross-sectional study. A longitudinal protocol is needed to investigate the relationship between anterior insula GMV, clinical symptoms and neuropsychological performance over time. Furthermore, such relationships should be contrasted along a trajectory of clinical course and functional impairment as proposed by McGorry, Hickie et al [49,50]. The insular cortex has also been implicated in addictive behaviour [108] and the relationship between anterior insula GMV and drug and alcohol use requires further investigation. Finally, whilst this present study found that a group of affective/psychosis patient exhibited reduced anterior insula GMV (Table 2), increases in anterior insula GMV correlating with increased symptom severity (Table 4) may apply to patients with autism spectrum disorder which have been shown to have enlarged insula GMVs relative to healthy controls [109].

## Conclusions

In summary, this study found that young people with emerging anxiety, affective or psychotic disorders have reduced left anterior insula GMV that is associated with poorer neurocognitive performance on an executive functioning task of set-shifting. Furthermore, patients with changes in right anterior insula GMV exhibited increased clinical symptom severity and more positive symptoms. By using the novel approach of examining a heterogeneous cohort of young depression, anxiety, bipolar and psychosis patients together, this study has demonstrated that insula GMV changes are associated with neurocognitive deficits and clinical symptoms in young patients. Future studies need to examine the longitudinal outcomes, clinical course and functional impairment of these findings, as well as their applicability to neurodevelopmental disorders.

## Abbreviations

ARMS = At-risk mental state; BPRS = Brief Psychiatric Rating Scale; COWAT = Controlled Oral Word Association Test; DASS = Depression Anxiety and Stress Scales; FMRI = Functional magnetic resonance imaging; GMV = Gray matter volume; HDRS = Hamilton Depression Rating Scale; ICV = Intracranial volume; IED = Intra-Dimensional/ Extra-Dimensional; K-10 = Kessler-10; LSAS = Liebowitz Social Anxiety Scale; MRI = Magnetic resonance imaging; ROI = Region of interest; RVP-A = Rapid Visual Information Processing A prime measure; SD = Standard deviation; SE = Standard error; SIAS = Social Interaction Anxiety Scale; SOFAS = Social and Occupational Functioning Assessment Scale; SPAI = Social Phobia and Anxiety Inventory; SPS = Social Phobia Scale; SSP = Spatial Span; TMT-B = Trail-Making Test part B; VBM = Voxel-based morphometry.

## Competing interests

The authors declare that they have no competing interests.

## Authors’ contributions

SNH produced the imaging analysis, statistical correlations and initial draft manuscript. IBH and MRB conceived of the study design and coordinated technical assistance. JL provided study design and interpretation of imaging analysis. DFH and SLN provided statistical analysis and interpretation of the clinical measures and neuropsychological tests. All authors contributed significantly to the interpretation of the data as well as having read and approved the final manuscript.

## Pre-publication history

The pre-publication history for this paper can be accessed here:

http://www.biomedcentral.com/1471-244X/12/45/prepub

## Supplementary Material

Additional file 1: Linear correlations of anterior insula GMV with clinical/neurocognitive variables controlling for years of education. Description of data: Linear correlations of clinical variables and neurocognitive executive functioning to patient anterior insula GMV, controlling for years of education. Pearson’s *r* (normal score distribution) or Spearman’s *ρ* (non-normal score distribution) correlation analysis explored the linear relationship between the left or right anterior insula GMV (corrected for ICV) against patient demographic, clinical variables or *z*-scores of neuropsychological assessments of executive function (*n* = 133), controlling for years of education. ^*^p < .05; ^**^p < .01 (2-tailed). Click here for file

## References

[B1] Augustine JR The insular lobe in primates including humans Neurological research 1985 7 2 10 2860583 10.1080/01616412.1985.11739692

[B2] Augustine JR Circuitry and functional aspects of the insular lobe in primates including humans Brain Res Brain Res Rev 1996 22 229 244 8957561 10.1016/s0165-0173(96)00011-2

[B3] Kober H Barrett LF Joseph J Bliss-Moreau E Lindquist K Wager TD Functional grouping and cortical-subcortical interactions in emotion: a meta-analysis of neuroimaging studies NeuroImage 2008 42 998 1031 10.1016/j.neuroimage.2008.03.059 18579414 PMC2752702

[B4] Damasio AR The feeling of what happens: body, emotion and the making of consciousness 2000 London: Vintage

[B5] Critchley HD Mathias CJ Dolan RJ Fear conditioning in humans: the influence of awareness and autonomic arousal on functional neuroanatomy Neuron 2002 33 653 663 10.1016/S0896-6273(02)00588-3 11856537

[B6] Jabbi M Bastiaansen J Keysers C A common anterior insula representation of disgust observation, experience and imagination shows divergent functional connectivity pathways PloS one 2008 3 e2939 10.1371/journal.pone.0002939 18698355 PMC2491556

[B7] Carr L Iacoboni M Dubeau MC Mazziotta JC Lenzi GL Neural mechanisms of empathy in humans: a relay from neural systems for imitation to limbic areas Proceedings of the National Academy of Sciences of the United States of America 2003 100 5497 5502 10.1073/pnas.0935845100 12682281 PMC154373

[B8] Takahashi H Matsuura M Koeda M Yahata N Suhara T Kato M Okubo Y Brain activations during judgments of positive self-conscious emotion and positive basic emotion: pride and joy Cerebral cortex 2008 18 898 903 17638925 10.1093/cercor/bhm120

[B9] Ortigue S Grafton ST Bianchi-Demicheli F Correlation between insula activation and self-reported quality of orgasm in women NeuroImage 2007 37 551 560 10.1016/j.neuroimage.2007.05.026 17601749

[B10] Winston JS Strange BA O’Doherty J Dolan RJ Automatic and intentional brain responses during evaluation of trustworthiness of faces Nature neuroscience 2002 5 277 283 10.1038/nn816 11850635

[B11] Michelon P Snyder AZ Buckner RL McAvoy M Zacks JM Neural correlates of incongruous visual information. An event-related fMRI study NeuroImage 2003 19 1612 1626 10.1016/S1053-8119(03)00111-3 12948716

[B12] Lamm C Singer T The role of anterior insular cortex in social emotions Brain structure & function 2010 214 579 591 10.1007/s00429-010-0251-3 20428887

[B13] Banati RB Goerres GW Tjoa C Aggleton JP Grasby P The functional anatomy of visual-tactile integration in man: a study using positron emission tomography Neuropsychologia 2000 38 115 124 10.1016/S0028-3932(99)00074-3 10660224

[B14] Bushara KO Grafman J Hallett M Neural correlates of auditory-visual stimulus onset asynchrony detection The Journal of neuroscience: the official journal of the Society for Neuroscience 2001 21 300 304 11150347 10.1523/JNEUROSCI.21-01-00300.2001 PMC6762435

[B15] Bagshaw MH Pribram KH Cortical organization in gustation (Macaca mulatta) Journal of neurophysiology 1953 16 499 508 13097198 10.1152/jn.1953.16.5.499

[B16] Penfield W Faulk ME The insula; further observations on its function Brain: a journal of neurology 1955 78 445 470 10.1093/brain/78.4.445 13293263

[B17] Sudakov K MacLean PD Reeves A Marino R Unit study of exteroceptive inputs to claustrocortex in awake, sitting, squirrel monkey Brain research 1971 28 19 34 10.1016/0006-8993(71)90521-X 4997565

[B18] Saper CB Convergence of autonomic and limbic connections in the insular cortex of the rat The Journal of comparative neurology 1982 210 163 173 10.1002/cne.902100207 7130477

[B19] Dupont S Bouilleret V Hasboun D Semah F Baulac M Functional anatomy of the insula: new insights from imaging Surgical and radiologic anatomy: SRA 2003 25 113 119 10.1007/s00276-003-0103-4 12819943

[B20] Flynn FG Benson DF Ardila A Anatomy of the insula - functional and clinical correlates Aphasiology 1999 13 55 78 10.1080/026870399402325

[B21] Mesulamm MM Mufson EJ Peters A, Jones E, Peters A, Jones E The Insula of Reil in man and monkey: architectonics, connectivity, and function Cerebral cortex 4 1985 New York: Plenum Press 179 226

[B22] Menon V Uddin LQ Saliency, switching, attention and control: a network model of insula function Brain structure & function 2010 214 655 667 10.1007/s00429-010-0262-0 20512370 PMC2899886

[B23] Cole MW Schneider W The cognitive control network: Integrated cortical regions with dissociable functions NeuroImage 2007 37 343 360 10.1016/j.neuroimage.2007.03.071 17553704

[B24] Dosenbach NU Fair DA Miezin FM Cohen AL Wenger KK Dosenbach RA Fox MD Snyder AZ Vincent JL Raichle ME Distinct brain networks for adaptive and stable task control in humans Proceedings of the National Academy of Sciences of the United States of America 2007 104 11073 11078 10.1073/pnas.0704320104 17576922 PMC1904171

[B25] Sridharan D Levitin DJ Menon V A critical role for the right fronto-insular cortex in switching between central-executive and default-mode networks Proceedings of the National Academy of Sciences of the United States of America 2008 105 12569 12574 10.1073/pnas.0800005105 18723676 PMC2527952

[B26] Ellison-Wright I Bullmore E Anatomy of bipolar disorder and schizophrenia: a meta-analysis Schizophr Res 2010 117 1 12 10.1016/j.schres.2009.12.022 20071149

[B27] Bora E Fornito A Yucel M Pantelis C Voxelwise meta-analysis of gray matter abnormalities in bipolar disorder Biol Psychiatry 2010 67 1097 1105 10.1016/j.biopsych.2010.01.020 20303066

[B28] Hallahan B Newell J Soares JC Brambilla P Strakowski SM Fleck DE Kieseppa T Altshuler LL Fornito A Malhi GS Structural magnetic resonance imaging in bipolar disorder: an international collaborative mega-analysis of individual adult patient data Biological psychiatry 2011 69 326 335 10.1016/j.biopsych.2010.08.029 21030008

[B29] Caetano SC Olvera RL Glahn D Fonseca M Pliszka S Soares JC Fronto-limbic brain abnormalities in juvenile onset bipolar disorder Biological psychiatry 2005 58 525 531 10.1016/j.biopsych.2005.04.027 16018982

[B30] Fornito A Yücel M Patti J Wood SJ Pantelis C Mapping grey matter reductions in schizophrenia: An anatomical likelihood estimation analysis of voxel-based morphometry studies Schizophrenia Research 2009 108 104 113 10.1016/j.schres.2008.12.011 19157788

[B31] Glahn DC Laird AR Ellison-Wright I Thelen SM Robinson JL Lancaster JL Bullmore E Fox PT Meta-analysis of gray matter anomalies in schizophrenia: application of anatomic likelihood estimation and network analysis Biol Psychiatry 2008 64 774 781 10.1016/j.biopsych.2008.03.031 18486104 PMC5441233

[B32] Bora E Fornito A Radua J Walterfang M Seal M Wood SJ Yucel M Velakoulis D Pantelis C Neuroanatomical abnormalities in schizophrenia: A multimodal voxelwise meta-analysis and meta-regression analysis Schizophrenia research 2011 127 46 57 10.1016/j.schres.2010.12.020 21300524

[B33] Olabi B Ellison-Wright I McIntosh AM Wood SJ Bullmore E Lawrie SM Are There Progressive Brain Changes in Schizophrenia? A Meta-Analysis of Structural Magnetic Resonance Imaging Studies Biological psychiatry 2011 70 88 96 10.1016/j.biopsych.2011.01.032 21457946

[B34] Bennett MR Schizophrenia: susceptibility genes, dendritic-spine pathology and gray matter loss Progress in neurobiology 2011 95 275 300 10.1016/j.pneurobio.2011.08.003 21907759

[B35] Critchley HD Wiens S Rotshtein P Ohman A Dolan RJ Neural systems supporting interoceptive awareness Nature neuroscience 2004 7 189 195 10.1038/nn1176 14730305

[B36] Shah SG Klumpp H Angstadt M Nathan PJ Phan KL Amygdala and insula response to emotional images in patients with generalized social anxiety disorder Journal of psychiatry & neuroscience: JPN 2009 34 296 302 PMC2702447 19568481

[B37] Rosso IM Makris N Britton JC Price LM Gold AL Zai D Bruyere J Deckersbach T Killgore WD Rauch SL Anxiety sensitivity correlates with two indices of right anterior insula structure in specific animal phobia Depression and anxiety 2010 27 1104 1110 10.1002/da.20765 21132846 PMC3010373

[B38] Kempton MJ Salvador Z Munafo MR Geddes JR Simmons A Frangou S Williams SC Structural Neuroimaging Studies in Major Depressive Disorder: Meta-analysis and Comparison With Bipolar Disorder Archives of general psychiatry 2011 68 675 690 10.1001/archgenpsychiatry.2011.60 21727252

[B39] Koolschijn PC van Haren NE Lensvelt-Mulders GJ Hulshoff Pol HE Kahn RS Brain volume abnormalities in major depressive disorder: a meta-analysis of magnetic resonance imaging studies Hum Brain Mapp 2009 30 3719 3735 10.1002/hbm.20801 19441021 PMC6871089

[B40] Arnone D McIntosh AM Ebmeier KP Munafo MR Anderson IM Magnetic resonance imaging studies in unipolar depression: Systematic review and meta-regression analyses European neuropsychopharmacology: the journal of the European College of Neuropsychopharmacology 2011 22 1 16 21723712 10.1016/j.euroneuro.2011.05.003

[B41] Bennett MR The prefrontal-limbic network in depression: A core pathology of synapse regression Progress in neurobiology 2011 93 457 467 10.1016/j.pneurobio.2011.01.001 21335052

[B42] Takahashi T Yucel M Lorenzetti V Tanino R Whittle S Suzuki M Walterfang M Pantelis C Allen N Volumetric MRI study of the insular cortex in individuals with current and past major depression J Affect Disord 2010 121 231 238 10.1016/j.jad.2009.06.003 19540599

[B43] Jarnum H Eskildsen SF Steffensen EG Lundbye-Christensen S Simonsen CW Thomsen IS Frund ET Theberge J Larsson EM Longitudinal MRI study of cortical thickness, perfusion, and metabolite levels in major depressive disorder Acta psychiatrica Scandinavica 2011 124 435 446 10.1111/j.1600-0447.2011.01766.x 21923809

[B44] Fusar-Poli P Borgwardt S Crescini A Deste G Kempton MJ Lawrie S Mc Guire P Sacchetti E Neuroanatomy of vulnerability to psychosis: a voxel-based meta-analysis Neuroscience and biobehavioral reviews 2011 35 1175 1185 10.1016/j.neubiorev.2010.12.005 21168439

[B45] Monkul ES Malhi GS Soares JC Anatomical MRI abnormalities in bipolar disorder: do they exist and do they progress? The Australian and New Zealand journal of psychiatry 2005 39 222 226 15777357 10.1080/j.1440-1614.2005.01571.x

[B46] Lagopoulos J Hermens DF Naismith SL Scott EM Hickie IB Frontal lobe changes occur early in the course of affective disorders in young people BMC psychiatry 2012 12 4 10.1186/1471-244X-12-4 22264318 PMC3280164

[B47] McGorry PD Hickie IB Yung AR Pantelis C Jackson HJ Clinical staging of psychiatric disorders: a heuristic framework for choosing earlier, safer and more effective interventions The Australian and New Zealand journal of psychiatry 2006 40 616 622 10.1080/j.1440-1614.2006.01860.x 16866756

[B48] McGorry PD Nelson B Amminger GP Bechdolf A Francey SM Berger G Riecher-Rossler A Klosterkotter J Ruhrmann S Schultze-Lutter F Intervention in individuals at ultra-high risk for psychosis: a review and future directions J Clin Psychiatry 2009 70 1206 1212 Epub 2009 Jun 1230 10.4088/JCP.08r04472 19573499

[B49] McGorry PD Nelson B Goldstone S Yung AR Clinical staging: a heuristic and practical strategy for new research and better health and social outcomes for psychotic and related mood disorders Canadian journal of psychiatry Revue canadienne de psychiatrie 2010 55 486 497 20723276 10.1177/070674371005500803

[B50] Hickie IB Scott EM Hermens DF Naismith SL Guastella AJ Kaur M Sidis A Whitwell B Glozier N Davenport T Applying clinical staging to young people who present for mental health care Early Intervention in Psychiatryin press 10.1111/j.1751-7893.2012.00366.x 22672533

[B51] Scott EM Hermens DF Glozier N Naismith SL Guastella AJ Hickie IB Targeted primary care-based mental health services engage young Australians in treatment Medical Journal of Australia 2012 196 136 140 10.5694/mja11.10481 22304610

[B52] Scott E Naismith S Whitwell B Hamilton B Chudleigh C Hickie I Delivering youth-specific mental health services: the advantages of a collaborative, multi-disciplinary system Australasian psychiatry: bulletin of Royal Australian and New Zealand College of Psychiatrists 2009 17 189 194 19296265 10.1080/10398560802657322

[B53] American Psychiatric Association Diagnostic criteria from DSM-IV-TR 2000 Washington, D.C: The Association

[B54] Hermens DF Redoblado Hodge MA Naismith SL Kaur M Scott E Hickie IB Neuropsychological clustering highlights cognitive differences in young people presenting with depressive symptoms Journal of the International Neuropsychological Society: JINS 2011 17 267 276 10.1017/S1355617710001566 21208478

[B55] Hermens DF Ward PB Hodge MA Kaur M Naismith SL Hickie IB Impaired MMN/P3a complex in first-episode psychosis: cognitive and psychosocial associations Progress in neuro-psychopharmacology & biological psychiatry 2010 34 822 829 10.1016/j.pnpbp.2010.03.019 20302901

[B56] Goldman HH Skodol AE Lave TR Revising axis V for DSM-IV: a review of measures of social functioning The American journal of psychiatry 1992 149 1148 1156 1386964 10.1176/ajp.149.9.1148

[B57] Hamilton M Development of a rating scale for primary depressive illness The British journal of social and clinical psychology 1967 6 278 296 10.1111/j.2044-8260.1967.tb00530.x 6080235

[B58] Overall JE Gorham DR The Brief Psychiatric Rating-Scale Psychological reports 1962 10 799 812 10.2466/pr0.1962.10.3.799

[B59] Kessler RC Andrews G Colpe LJ Hiripi E Mroczek DK Normand SL Walters EE Zaslavsky AM Short screening scales to monitor population prevalences and trends in non-specific psychological distress Psychological medicine 2002 32 959 976 10.1017/S0033291702006074 12214795

[B60] Andrews G Slade T Interpreting scores on the Kessler Psychological Distress Scale (K10) Australian and New Zealand journal of public health 2001 25 494 497 10.1111/j.1467-842X.2001.tb00310.x 11824981

[B61] Lovibond PF Lovibond SH The structure of negative emotional states: comparison of the Depression Anxiety Stress Scales (DASS) with the Beck Depression and Anxiety Inventories Behaviour research and therapy 1995 33 335 343 10.1016/0005-7967(94)00075-U 7726811

[B62] Mattick RP Clark JC Development and validation of measures of social phobia scrutiny fear and social interaction anxiety 1989 Australia: University of New South Wales Unpublished manuscript edition: Unpublished manuscript 10.1016/s0005-7967(97)10031-6 9670605

[B63] Hermens DF Naismith SL Redoblado Hodge MA Scott EM Hickie IB Impaired verbal memory in young adults with unipolar and bipolar depression Early intervention in psychiatry 2010 4 227 233 10.1111/j.1751-7893.2010.00194.x 20712728

[B64] Wechsler D Wechsler Test of Adult Reading 2001 San Antonio, TX: Psychological Corporation

[B65] Strauss E Sherman EMS Spreen O A compendium of neuropsychological tests : administration, norms, and commentary 2006 3 Oxford ; New York: Oxford University Press

[B66] Tombaugh TN, Kozak J, Rees L Normative data for the controlled oral word association test 1996 New York: Oxford University Press

[B67] Sahakian BJ Owen AM Computerized assessment in neuropsychiatry using CANTAB: discussion paper Journal of the Royal Society of Medicine 1992 85 399 402 1629849 PMC1293547

[B68] Dale A Fischl B Sereno MI Cortical Surface-Based Analysis: I. Segmentation and Surface Reconstruction Neuroimage 1999 9 179 194 10.1006/nimg.1998.0395 9931268

[B69] Fischl B Dale AM Measuring the thickness of the human cerebral cortex from magnetic resonance images Proceedings of the National Academy of Sciences of the United States of America 2000 97 11050 11055 10.1073/pnas.200033797 10984517 PMC27146

[B70] Fischl B Liu A Dale AM Automated manifold surgery: constructing geometrically accurate and topologically correct models of the human cerebral cortex IEEE Medical Imaging 2001 20 70 80 10.1109/42.906426 11293693

[B71] Fischl B Salat DH Busa E Albert M Dieterich M Haselgrove C van der Kouwe A Killiany R Kennedy D Klaveness S Whole brain segmentation: automated labeling of neuroanatomical structures in the human brain Neuron 2002 33 341 355 10.1016/S0896-6273(02)00569-X 11832223

[B72] Fischl B Salat DH van der Kouwe AJW Makris N Ségonne F Quinn BT Dale AM Sequence-independent segmentation of magnetic resonance images Neuroimage 2004 23 S69 S84 15501102 10.1016/j.neuroimage.2004.07.016

[B73] Fischl B Sereno MI Dale AM Cortical surface-based analysis. II: Inflation, flattening, and a surface-based coordinate system NeuroImage 1999 9 195 207 10.1006/nimg.1998.0396 9931269

[B74] Fischl B Sereno MI Tootell RB Dale AM High-resolution intersubject averaging and a coordinate system for the cortical surface Human brain mapping 1999 8 272 284 10.1002/(SICI)1097-0193(1999)8:4<272::AID-HBM10>3.0.CO;2-4 10619420 PMC6873338

[B75] Fischl B van der Kouwe A Destrieux C Halgren E Ségonne F Salat DH Busa E Seidman LJ Goldstein J Kennedy D Automatically Parcellating the Human Cerebral Cortex Cerebral Cortex 2004 14 11 22 10.1093/cercor/bhg087 14654453

[B76] Han X Jovicich J Salat D van der Kouwe A Quinn B Czanner S Busa E Pacheco J Albert M Killiany R Reliability of MRI-derived measurements of human cerebral cortical thickness: The effects of field strength, scanner upgrade and manufacturer Neuroimage 2006 32 180 194 10.1016/j.neuroimage.2006.02.051 16651008

[B77] Jovicich J Czanner S Greve D Haley E van der Kouwe A Gollub R Kennedy D Schmitt F Brown G MacFall J Reliability in multi-site structural MRI studies: Effects of gradient non-linearity correction on phantom and human data Neuroimage 2006 30 436 443 10.1016/j.neuroimage.2005.09.046 16300968

[B78] Segonne F Dale AM Busa E Glessner M Salat D Hahn HK Fischl B A hybrid approach to the skull stripping problem in MRI Neuroimage 2004 22 1060 1075 10.1016/j.neuroimage.2004.03.032 15219578

[B79] Reuter M Rosas HD Fischl B Highly Accurate Inverse Consistent Registration: A Robust Approach Neuroimage 2010 53 1181 1196 10.1016/j.neuroimage.2010.07.020 20637289 PMC2946852

[B80] Sled JG Zijdenbos AP Evans AC A nonparametric method for automatic correction of intensity nonuniformity in MRI data IEEE Trans Med Imaging 1998 17 87 97 10.1109/42.668698 9617910

[B81] Segonne F Pacheco J Fischl B Geometrically accurate topology-correction of cortical surfaces using nonseparating loops IEEE Trans Med Imaging 2007 26 518 529 17427739 10.1109/TMI.2006.887364

[B82] Dale AM Sereno MI Improved localization of cortical activity by combining EEG and MEG with MRI cortical surface reconstruction: a linear approach J Cogn Neurosci 1993 5 162 176 10.1162/jocn.1993.5.2.162 23972151

[B83] Rosas HD Liu AK Hersch S Glessner M Ferrante RJ Salat DH van der Kouwe A Jenkins BG Dale AM Fischl B Regional and progressive thinning of the cortical ribbon in Huntington’s disease Neurology 2002 58 695 701 10.1212/WNL.58.5.695 11889230

[B84] Kuperberg GR Broome MR McGuire PK David AS Eddy M Ozawa F Goff D West WC Williams SC van der Kouwe AJ Regionally localized thinning of the cerebral cortex in schizophrenia Archives of general psychiatry 2003 60 878 888 10.1001/archpsyc.60.9.878 12963669

[B85] Salat D Buckner RL Snyder AZ Greve DN Desikan RS Busa E Morris JC Dale A Fischl B Thinning of the cerebral cortex in aging Cerebral Cortex 2004 14 721 730 10.1093/cercor/bhh032 15054051

[B86] Destrieux C Fischl B Dale A Halgren E Automatic parcellation of human cortical gyri and sulci using standard anatomical nomenclature NeuroImage 2010 53 1 15 10.1016/j.neuroimage.2010.06.010 20547229 PMC2937159

[B87] Buckner RL Head D Parker J Fotenos AF Marcus D Morris JC Snyder AZ A unified approach for morphometric and functional data analysis in young, old, and demented adults using automated atlas-based head size normalization: reliability and validation against manual measurement of total intracranial volume NeuroImage 2004 23 724 738 10.1016/j.neuroimage.2004.06.018 15488422

[B88] Naismith S Hickie I Ward PB Turner K Scott E Little C Mitchell P Wilhelm K Parker G Caudate nucleus volumes and genetic determinants of homocysteine metabolism in the prediction of psychomotor speed in older persons with depression The American journal of psychiatry 2002 159 2096 2098 10.1176/appi.ajp.159.12.2096 12450963

[B89] Carpenter MB Core text of neuroanatomy 1991 4 Baltimore: Williams & Wilkins

[B90] Millan MJ Agid Y Brune M Bullmore ET Carter CS Clayton NS Connor R Davis S Deakin B Derubeis RJ Cognitive dysfunction in psychiatric disorders: characteristics, causes and the quest for improved therapy Nature reviews Drug discovery 2012 11 141 168 10.1038/nrd3628 22293568

[B91] Paulus MP Stein MB Interoception in anxiety and depression Brain structure & function 2010 214 451 463 10.1007/s00429-010-0258-9 20490545 PMC2886901

[B92] Paulus MP Stein MB An insular view of anxiety Biological psychiatry 2006 60 383 387 10.1016/j.biopsych.2006.03.042 16780813

[B93] Stein MB Simmons AN Feinstein JS Paulus MP Increased amygdala and insula activation during emotion processing in anxiety-prone subjects The American journal of psychiatry 2007 164 318 327 10.1176/appi.ajp.164.2.318 17267796

[B94] Nagai M Kishi K Kato S Insular cortex and neuropsychiatric disorders: a review of recent literature European psychiatry: the journal of the Association of European Psychiatrists 2007 22 387 394 10.1016/j.eurpsy.2007.02.006 17416488

[B95] Sommer IE Diederen KM Blom JD Willems A Kushan L Slotema K Boks MP Daalman K Hoek HW Neggers SF Kahn RS Auditory verbal hallucinations predominantly activate the right inferior frontal area Brain: a journal of neurology 2008 131 3169 3177 10.1093/brain/awn251 18854323

[B96] Shapleske J Rossell SL Chitnis XA Suckling J Simmons A Bullmore ET Woodruff PW David AS A computational morphometric MRI study of schizophrenia: effects of hallucinations Cereb Cortex 2002 12 1331 1341 10.1093/cercor/12.12.1331 12427683

[B97] Craig AD How do you feel–now? The anterior insula and human awareness Nature reviews Neuroscience 2009 10 59 70 10.1038/nrn2555 19096369

[B98] Heimberg RG Mueller GP Holt CS Hope DA Liebowitz MR Assessment of anxiety in social interaction and being observed by others: the social interaction anxiety scale and the social phobia scale Behavior Therapy 1992 23 53 75 10.1016/S0005-7894(05)80308-9

[B99] Liebowitz MR Social phobia Modern problems of pharmacopsychiatry 1987 22 141 173 2885745 10.1159/000414022

[B100] Turner SM Beidel DC Dancu CV Stanley MA An empirically derived inventory to measure social fears and anxiety: the Social phobia and Anxiety Inventory Psychological Assessment 1989 1 35 40

[B101] Turner SM Beidel DC Dancu C Stanley MA Social Phobia and Anxiety Inventory Manual 1996 North Tonawanda, NY: Multihealth Systems

[B102] Carleton RN Collimore KC Asmundson GJ McCabe RE Rowa K Antony MM Refining and validating the Social Interaction Anxiety Scale and the Social Phobia Scale Depression and anxiety 2009 26 E71 E81 10.1002/da.20480 19152346

[B103] Hunsberger J Austin DR Henter ID Chen G The neurotrophic and neuroprotective effects of psychotropic agents Dialogues in clinical neuroscience 2009 11 333 348 19877500 10.31887/DCNS.2009.11.3/jhunsberger PMC2804881

[B104] Shepherd AM Laurens KR Matheson SL Carr VJ Green MJ Systematic meta-review and quality assessment of the structural brain alterations in schizophrenia Neuroscience and biobehavioral reviews 2012 36 1342 1356 10.1016/j.neubiorev.2011.12.015 22244985

[B105] Ho BC Andreasen NC Ziebell S Pierson R Magnotta V Long-term antipsychotic treatment and brain volumes: a longitudinal study of first-episode schizophrenia Archives of general psychiatry 2011 68 128 137 10.1001/archgenpsychiatry.2010.199 21300943 PMC3476840

[B106] Takahashi T Wood S Soulsby B Tanino R Wong M McGorry P Suzuki M Velakoulis D Pantelis C Diagnostic specificity of the insular cortex abnormalities in first-episode psychotic disorders Prog Neuropsychopharmacol Biol Psychiatry 2009 33 651 657 10.1016/j.pnpbp.2009.03.005 19298837

[B107] Takahashi T Malhi GS Wood SJ Yücel M Walterfang M Tanino R Suzuki M Pantelis C Insular cortex volume in established bipolar affective disorder: A preliminary MRI study Psychiatry Research: Neuroimaging 2010 182 187 190 10.1016/j.pscychresns.2010.01.006 20417066

[B108] Naqvi NH Bechara A The insula and drug addiction: an interoceptive view of pleasure, urges, and decision-making Brain structure & function 2010 214 435 450 10.1007/s00429-010-0268-7 20512364 PMC3698865

[B109] Cauda F Geda E Sacco K D’Agata F Duca S Geminiani G Keller R Grey matter abnormality in autism spectrum disorder: an activation likelihood estimation meta-analysis study Journal of neurology, neurosurgery, and psychiatry 2011 82 1304 1313 10.1136/jnnp.2010.239111 21693631

