# Supplementary material for: Correlating anterior insula gray matter volume changes in young people with clinical and neurocognitive outcomes: an MRI study
Source: BMC Psychiatry. 2012 May 20;12:45. doi: 10.1186/1471-244X-12-45 (PMC3468394; doi:10.1186/1471-244X-12-45)
Supplement: Additional file 1: — Linear correlations of anterior insula GMV with clinical/neurocognitive variables controlling for years of education. Description of data: Linear correlations of clinical variables and neurocognitive executive functioning to patient anterior insula GMV, controlling for years of education. Pearson’s r (normal score distribution) or Spearman’s ρ (non-normal score distribution) correlation analysis explored the linear relationship between the left or right anterior insula GMV (corrected for ICV) against patient demographic, clinical variables or z-scores of neuropsychological assessments of executive function (n = 133), controlling for years of education. *p < .05; **p < .01 (2-tailed). [file 1471-244X-12-45-S1.doc]

# Additional File 1

**Linear correlations of anterior insula GMV with clinical or neurocognitive variables controlling for years of education**

| **Demographic/clinical variable** | **Left anterior insula** | **Right anterior insula** |
| --- | --- | --- |
| Predicted IQ | *r*(115)= .07 | *r*(115)= .01 |
| Age of onset of illness | *r*(120)= -.03 | *r*(120)= -.06 |
| Antidepressant dose (mg/day) | *r*(60)= .09 | *r*(60)= -.09 |
| Antipsychotic dose (mg/day) | *r*(58)= .14 | *r*(58)= .12 |
| Mood stabiliser dose (mg/day) | *r*(23)= -.10 | *r*(23)= .08 |
| Social and occupational functioning (SOFAS) | *r*(114)= -.09 | *r*(114)= -.10 |
| Psychological distress (K-10) | *r*(123)= .08 | *r*(123)= .07 |
| Depression (HDRS) | *r*(119)= .11 | *r*(119)= -.03 |
| Depression (DASS Depression subscore) | *r*(115)= .10 | *r*(115)= -.00 |
| Anxiety (DASS Anxiety subscore) | *r*(115)= -.02 | *r*(115)= -.04 |
| Stress (DASS Stress subscore) | *r*(115)= .05 | *r*(115)= .01 |
| Social interaction anxiety (SIAS) | *r*(119)= .19^*^ | *r*(119)= .24^**^ |
| Symptom severity (BPRS Total) | *r*(115)= .08 | *r*(115)= -.02 |
| Positive symptoms (BPRS Positive Symptoms subscore) | *r*(115)= .03 | *r*(115)= .03 |
| Negative symptoms (BPRS Negative Symptoms subscore) | *r*(115)= .10 | *r*(115)= .01 |
| Depression (BPRS Depression subscore) | *r*(115)= .03 | *r*(115)= -.07 |
| Mania (BPRS Mania subscore) | *r*(114)= .09 | *r*(114)= .06 |
| Set Shifting (IED Total error) | *ρ*(109)= .32^**^ | *ρ*(109)= .13 |
| Working Memory (SSP) | *ρ*(107)= .08 | *ρ*(107)= -.00 |
| Mental Flexibility (TMT-B) | *ρ*(114)= -.04 | *ρ*(114)= .04 |
| Sustained Attention (RVP-A) | *r*(102)= .03 | *r*(102)= .13 |
| Verbal Fluency (COWAT) | *r*(107)= .10 | *r*(107)= .09 |

Pearson’s *r* (normal score distribution) or Spearman’s *ρ* (non-normal score distribution) correlation analysis explored the linear relationship between the left or right anterior insula GMV (corrected for ICV) against patient demographic, clinical variables or *z*-scores of neuropsychological assessments of executive function (*n*=133), controlling for years of education. ^*^ *p* < .05; ^**^ *p* < .01 (2-tailed).
